# The effect of dietary tryptophan supplementation, and an oral tryptophan challenge, on urinary excretion of 5-hydroxyindoleacetic acid in domestic dogs

**DOI:** 10.3389/fvets.2025.1634940

**Published:** 2025-09-29

**Authors:** Chloe Cheung, Jess Rigling, Bernadette Stang, Craig Ruaux

**Affiliations:** ^1^University Veterinary Teaching Hospital Sydney, Sydney School of Veterinary Science, The University of Sydney, Sydney, NSW, Australia; ^2^Veterinary Clinical Sciences, Oregon State University, Corvallis, OR, United States

**Keywords:** serotonin, tryptophan, chronic enteropathy, inflammatory bowel disease, 5-hydroxyindoleacetic acid

## Abstract

Chronic enteropathies, commonly referred to as inflammatory bowel disease, are multi-factorial disorders that affect a substantial proportion of both the human population and companion animals. There is an emerging body of evidence suggesting that alterations in serotonin metabolism may contribute to the development of inflammatory bowel disease in humans. 5-Hydroxyindole acetic acid (5-HIAA) is a major metabolite of serotonin which undergoes renal excretion, providing a non-invasive indicator of serotonin metabolism. This study validated a commercial ELISA assay for 5-HIAA for use in canines and investigated the effect of dietary tryptophan supplementation and tryptophan challenge on the excretion of 5-HIAA in healthy dogs (*n* = 14). Dietary supplementation was associated with sustained alterations in serotonin metabolism, whereas a short-term oral tryptophan challenge, did not significantly impact immediate 5-HIAA excretion at 4- and 8-h post-challenge. These findings provide key insights regarding potential confounding factors for the interpretation of urinary 5-HIAA secretion as a marker of serotonin metabolism in domestic animals. Future prospective studies with a larger sample size are required to compare the serotonin concentrations between dogs with chronic enteropathy and healthy dogs on the tryptophan loading/challenge tests.

## Introduction

Chronic enteropathy (CE) is a collective medical term for any gastrointestinal condition that has persisted for over 3 weeks (1). Typical clinical signs include vomiting, diarrhea, anorexia, and chronic weight loss. Once other gastrointestinal disorders, such as infections, obstructions, and extra-gastrointestinal diseases, such as hypoadrenocorticism, pancreatopathies, and neoplasia are excluded, idiopathic inflammatory bowel disease (IBD) can be presumptively diagnosed (2). In humans, growing evidence suggests that altered serotonin (5-hydroxytryptamine, 5-HT) metabolism contributes to IBD pathogenesis. Plasma and urine 5-HT concentrations have been studied in dogs with myxomatous mitral valve disease and in veterinary behavioral medicine (3–5). Limited data exists on the role of 5-HT in dogs with CE. A previous study demonstrated a significant increase in 5-HT and chromogranin-A in the gastrointestinal mucosa of dogs with CE compared with healthy controls (6).

5-Hydroxyindole acetic acid (5-HIAA) is a stable metabolite of serotonin metabolism which is excreted via a renal pathway and may serve as a stable, non-invasive marker of 5-HT turnover. Tryptophan (TRP) is an essential amino acid mainly found in dietary proteins and is the sole precursor for 5-HT production (27). Decreased serum concentrations of TRP has been documented in human with IBD (7, 8). TRP supplementation improved clinical signs of experimentally induced colitis and could be a promising therapeutic option for some IBD patients (9, 10). In veterinary medicine, most previous studies have focused on the effects of TRP supplementation on animal behavior (11, 12). Urinary 5-HIAA was selected for analysis over serum 5-HIAA as it is a stable metabolite of 5-HT, readily measurable with a commercially available ELISA, and provides an integrated measure of 5-HT turnover over time.

The aims of the study were: (1) to validate a commercially ELISA assay for canine urinary 5-HIAA, and (2) to evaluate the effects of oral TRP supplementation and oral TRP challenge on urinary 5-HIAA-to-creatinine ratio (UH: CR) in healthy dogs. We hypothesized that UH: CR would increase following dietary TRP supplementation and an oral TRP challenge would produce a peak UH: CR at 4 h post-dosing.

## Materials and methods

### Urine 5-HIAA assay validation

The urinary 5-HIAA assay validation was performed by three of the authors (JR, BS, CR) at Oregon State University College of Veterinary Medicine. Urinary 5-HIAA assay kits were obtained from IBL-America. Voided urine samples were collected from 20 apparently healthy dogs owned by students, staff, and faculty. Samples were protected from light by wrapping the sample tubes in foil and refrigerated at 0 to 4 °C if processing occurred more than 30 min after collection. The urine samples were first divided into two aliquots - one aliquot was acidified to a pH of less than 4 by dropwise addition of 6 M hydrochloric acid, using pH paper to gage the effect, and was later stored at −20 °C for future batch analysis; the other, non-acidified, aliquot was submitted to the in-house pathology service for urine creatinine measurement.

The acidified urine samples were first thawed to room temperature before processing. To assess the potential matrix effect from the canine urine, multiple urine samples were pooled to provide a larger volume, and replicates (*n* = 3) of a serial dilution series, 1:1, 1:2, 1:4, and 1:8, were performed using the pooled urine, by admixing the assay buffer solution with the pooled urine volume.

Accuracy was assessed by measurement of recovery of pure 5-HIAA added in known, varying quantities to acidified urine samples of varying specific gravities (*n* = 3 urine samples, three replicates/spike). Precision was assessed via measurement of inter- and intra-assay variation. Multiple replicates (*n* = 8) of each of the three samples of varying specific gravity were assessed within an individual plate to obtain the intra-assay variability; the same three urine samples used for intra-assay variability were assayed on every plate run throughout the study to determine the inter-assay variability.

### Tryptophan supplementation study design

This component of the study used a cross-sectional prospective design to assess the influence of TRP supplementation on urine 5-HIAA excretion in healthy dogs on a standardized diet. This was a non-blinded, internally controlled cross-over study. All participating dogs were volunteered by staff members at the University Veterinary Teaching Hospital Sydney between March 2024 and May 2024. The University of Sydney Animal Ethics Committee approved this study (Protocol 2023/2379). Participation involved two distinct study phases—a dietary TRP supplementation trial and an oral TRP challenge.

All participating dogs underwent health screening, including detailed history collection and physical examination. They were all judged to be clinically healthy and were not receiving any treatment or supplements; however routine blood work was not performed. Any dogs with historical pre-existing medical conditions or who were unable to complete their participation in the study were excluded from the final analysis. Dogs that had recently received glucocorticoids or any medications that may alter serotonin metabolism, such as tricyclic antidepressants, opioids, antiepileptics, and monoamine oxidase inhibitors, were excluded from participation.

All dogs received Purina Pro Plan Adult Sensitive Skin & Stomach for small/medium & large breeds dry food strictly in the recommended amount according to the feeding guidelines by the manufacturer for 2 weeks. Diet for this study was generously donated by Nestlé-Purina Petcare Australia.

After receiving informed owner consent and enrollment in the study, all dogs underwent a two-week dietary transition and washout period to the standardized Sensitive Skin and Stomach Diet. The first urine sample (T_0_) was collected after the two-week period. The dogs then received a daily oral tryptophan supplementation (Paw by Blackmores Complete Calm™) for 1 week, using the recommended dosing regimen for their body weight according to the manufacturer (0–4 kg: ½ chew, 5–14 kg: 1 chew, 15–29 kg: 2 chews, 30 kg and above: 3 chews). The supplement was administered by the owner at home at regular times daily based on provided written dosing instructions and compliance was assessed by owner attestation at the time of sample collection. A second urine sample (T_1_) was then collected the day after the final TRP supplementation dose.

After completion of the dietary supplementation phase, oral TRP was discontinued, and all dogs underwent a 2-week washout period during which they remained exclusively on the standardized diet.

For the second part of the study the participating dogs were admitted to the University Veterinary Teaching Hospital Sydney in the morning. All the dogs were housed in a similar environment in a confined designated dog run separated from the populated hospital area, with free access to water. A baseline urine sample (T2_a_) was obtained once admitted into hospital. Dogs were given twice the manufacturer’s recommended dose of the tryptophan supplement orally (0–4 kg: 1 chew, 5–14 kg: 2 chews, 15–29 kg: 4 chews, 30 kg and above: 6 chews) immediately after the baseline urine was sampled. Subsequent urine samples were collected at 4 and 8 h (T2_b_ and T2_c_) after the oral tryptophan challenge, which concluded the dog’s participation in the study. All dogs had free access in a confined dog yard at 0, 4, and 8 h after the oral TRP challenge to facilitate voided urine sampling. No food was provided during this study period.

### Sample analysis—dietary supplementation trial

Voided urine was used for the urine 5-HIAA measurement. The urine was collected by the authors (CC, CR) in the hospital setting or by the owner at home if the participating dogs were excessively anxious in the hospital setting. The urine was then divided into three separate 1 mL microtubes, and 100 μL of 2 M hydrochloric acid was added to each microtube for acidification before storage. The acidified urine was thoroughly mixed with a vortex mixer at 1000 RPM for 10 s and stored at −80 °C for future batch analysis. The stored samples were later processed as per the protocol described above under assay validation.

All urine samples were thawed to room temperature before processing. Each step was followed closely according to the manufacturer’s protocol, and all samples were run in duplicate. The light absorbance of the ELISA plate was read by a Dynamica Halo LED 96 microplate reader at a wavelength of 450 nm. The urine 5-HIAA concentrations of the respective samples were later interpolated from the ELISA standard curve.

Matching thawed aliquots of non-acidified urine were submitted to the Sydney School of Veterinary Science clinical pathology service, Veterinary Pathology Diagnostic Services (VPDS), for batch urine creatinine analysis using a Thermo Konelab 30 Prime Chemistry Analyzer.

Reported urine 5-HIAA concentrations were multiplied by 8 to correct for the necessary dilution factor derived during the assay validation study. The urine 5-HIAA to creatinine ratio (UH: CR) was then calculated manually by dividing the urine 5-HIAA concentration by the urine creatinine concentration. Urine creatinine was used to control for variations in urine specific gravity, which is highly influenced by fluid intake and amount of physical activity.

### Data analysis

Statistical analysis was performed using GraphPad Prism v10.0 (GraphPad Software, Boston, MA, United States). All data sets were assessed for normality; for data sets that were not normally distributed, the Friedman test (a non-parametric repeated measures ANOVA equivalent) was used. For all analyses, a *p*-value of less than 0.05 was set as the threshold for statistical significance.

## Results

### Urine 5-HIAA validation

#### Matrix effect

**Figure 1:**
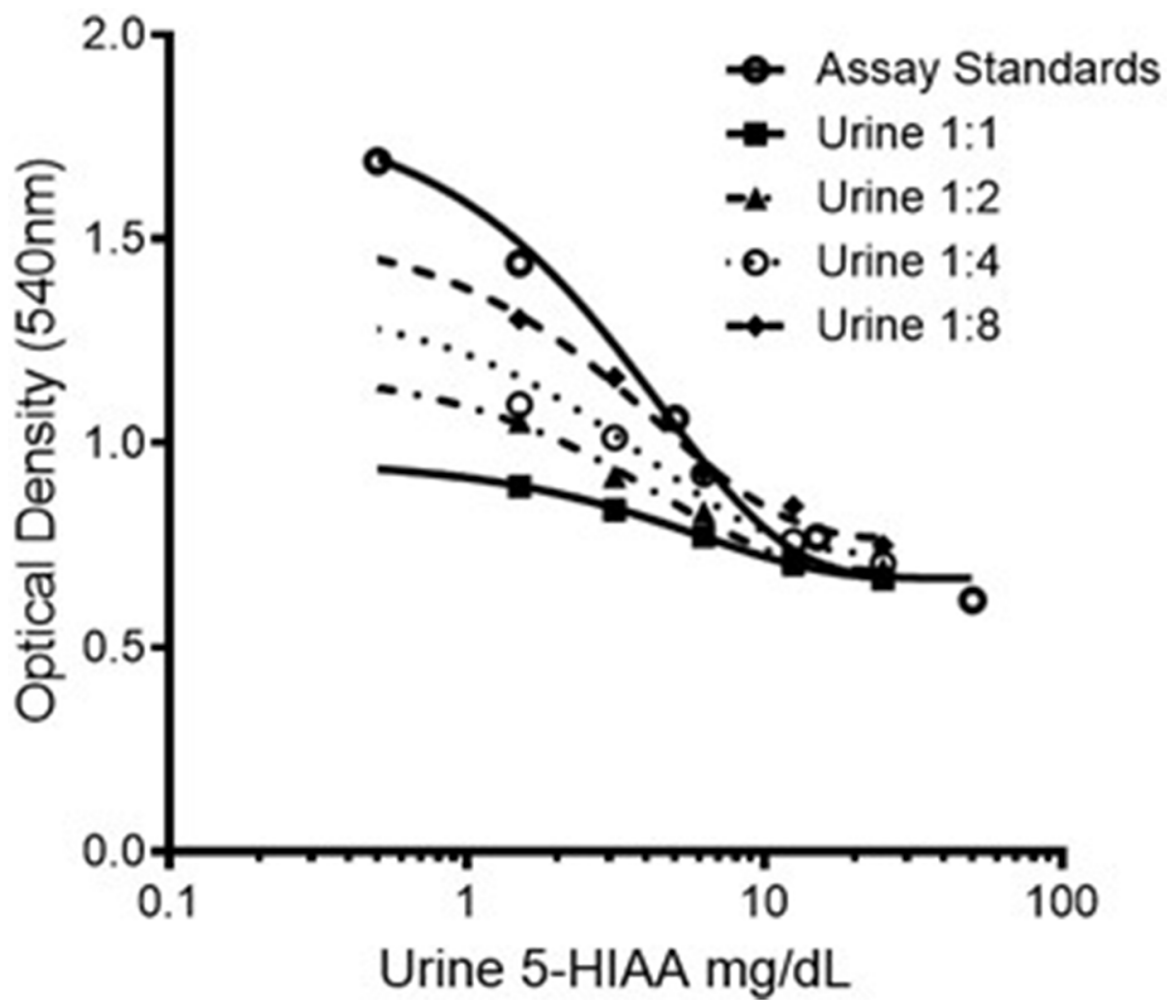
Representative dilution series of 5-HIAA ELISA standard showing varying chromogenic response with varying dilutions of pooled canine urine. 5-HIAA, 5-hydroxyindoleacetic acid.

A marked matrix effect from canine urine was noted, as illustrated in Figure 1. The use of undiluted canine urine as a sample matrix was associated with markedly reduced color formation in the chromogenic step of the assay, thus resulting in dramatic overestimation of 5-HIAA concentrations in dilutional series and low concentration spiking recovery experiments. This effect was mitigated by diluting the canine urine samples 1:8 in assay buffer as the first step of our assay protocol. Construction of standard curves using canine urine as the standard diluent was ruled out as this approach resulted in a marked loss of assay dynamic range.

For all subsequent results, values were derived from urine samples diluted in assay buffer 1:8 before the methylation step. According to the manufacturer’s instructions, this dilution step is not necessary for human-origin urine samples but, based on the data presented here, dilution is necessary when performing the assay with canine urine samples.

#### Assay precision and accuracy

Table 1 shows the dilutional parallelism of 5-HIAA in three pooled canine urine samples of varying specific gravity. The overall average of the observed:expected ratios for canine urine samples was 110.0%, starting from an undiluted sample. Spiking recovery of pure 5-HIAA added to three different urine samples in amounts ranging from 1.5 to 25 mg/L ranged from 118.87 to 143.6%, with an overall average spiking recovery of 132.66%.

**Table 1:** Dilutional parallelism *of* 5-HIAA in three pooled canine urine samples of varying specific gravity.

Dilution	Sample 1		Sample 2		Sample 3		Average % O/E
	Obs.	% O/E	Obs.	% O/E	Obs.	% O/E	
1:1	15.01	*NA*	25.34	*NA*	10.68	*NA*	*NA*
1:2	9.61	128.01	12.94	102.12	7.69	144.09	124.74
1:4	4.91	102.35	8.02	123.92	3.66	95.16	107.15
1:8	2.45	99.42	5.58	139.17	2.38	130.22	122.93
1:16	1.06	87.02	1.80	64.78	1.07	90.36	80.73

Samples were diluted 1:8 with assay buffer before analysis. 5-HIAA, 5-hydroindoleacetic acid; Obs, observation; O/E, observed-to-expected ratio; NA, not applicable.

The coefficients of variation for intra- and inter-assay variation, derived from three samples assayed 8 x each on a single plate, and the same three samples measured 8 x each on separate plates, ranged from 3.45–5.19% and 4.61–7.27% for intra- and inter-assay variability, respectively.

### The effect of dietary tryptophan loading on urinary 5-HIAA excretion

**Figure 2:**
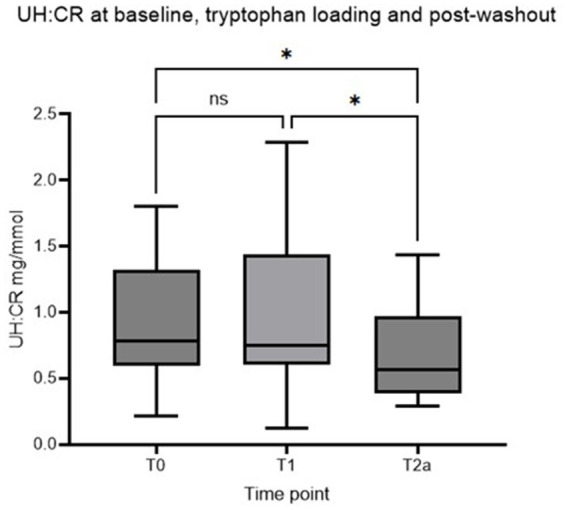
Box plot of UH: CR (mg/mmol) of healthy controls at T0, T1, and T2a, respectively (Asterisk* indicates statistical significance at *p* < 0.05. NS indicates no statistically significant difference). UH: CR, urinary 5-HIAA-to-creatinine ratio.

Sixteen staff dogs who met the inclusion criteria were recruited initially. Two dogs were later excluded as they were not consistently receiving the standardized diet. The remaining 14 dogs completed the whole duration of the study. Four neutered males and 10 neutered females were recruited. A variety of breeds were represented with ages ranging from 2.7 to 9.9 years (Median age: 5.1 years). Body weights ranged from 3.7 to 37.2 kg (Median body weight: 16.7 kg). Table 2 shows descriptive statistics for UH: CR after 2 weeks of diet transition (T_0_), after a week of TRP loading (T_1_), and at the end of the post-washout period (T_2a_). UH: CR was numerically higher at T_1_ compared with T_0_, but this difference did not reach statistical significance (*p* > 0.05). Figure 2 shows that UH: CR was significantly different between T_0_ and T_2a_, *p* = 0.025 (*p* < 0.05), and between T_1_ and T_2a_, *p* = 0.042 (*p* < 0.05). These suggest a week of TRP loading has sustained metabolic impact on 5-HT metabolism. The within-group variability in UH: CR was notably decreased at T_2a_ compared to T_1_.

**Table 2:** Descriptive statistics of UH: CR (mg/mmol) after 2 weeks of diet transition (T_0_), after TRP loading (T_1_) and post-washout period (T_2a_).

Time point	n	Mean UH: CR ± SD (mg/mmol)	CV (%)
T_0_	14	0.918 ± 0.483	52.59
T_1_	14	0.970 ± 0.639	65.82
T_2a_	14	0.669 ± 0.343	51.23

UH: CR, urinary 5-HIAA-to-creatinine ratio; TRP, tryptophan; SD, standard deviation; CV, coefficient of variation; n, number of dogs.

### The effect of an oral tryptophan challenge on urinary 5-HIAA excretion

**Figure 3:**
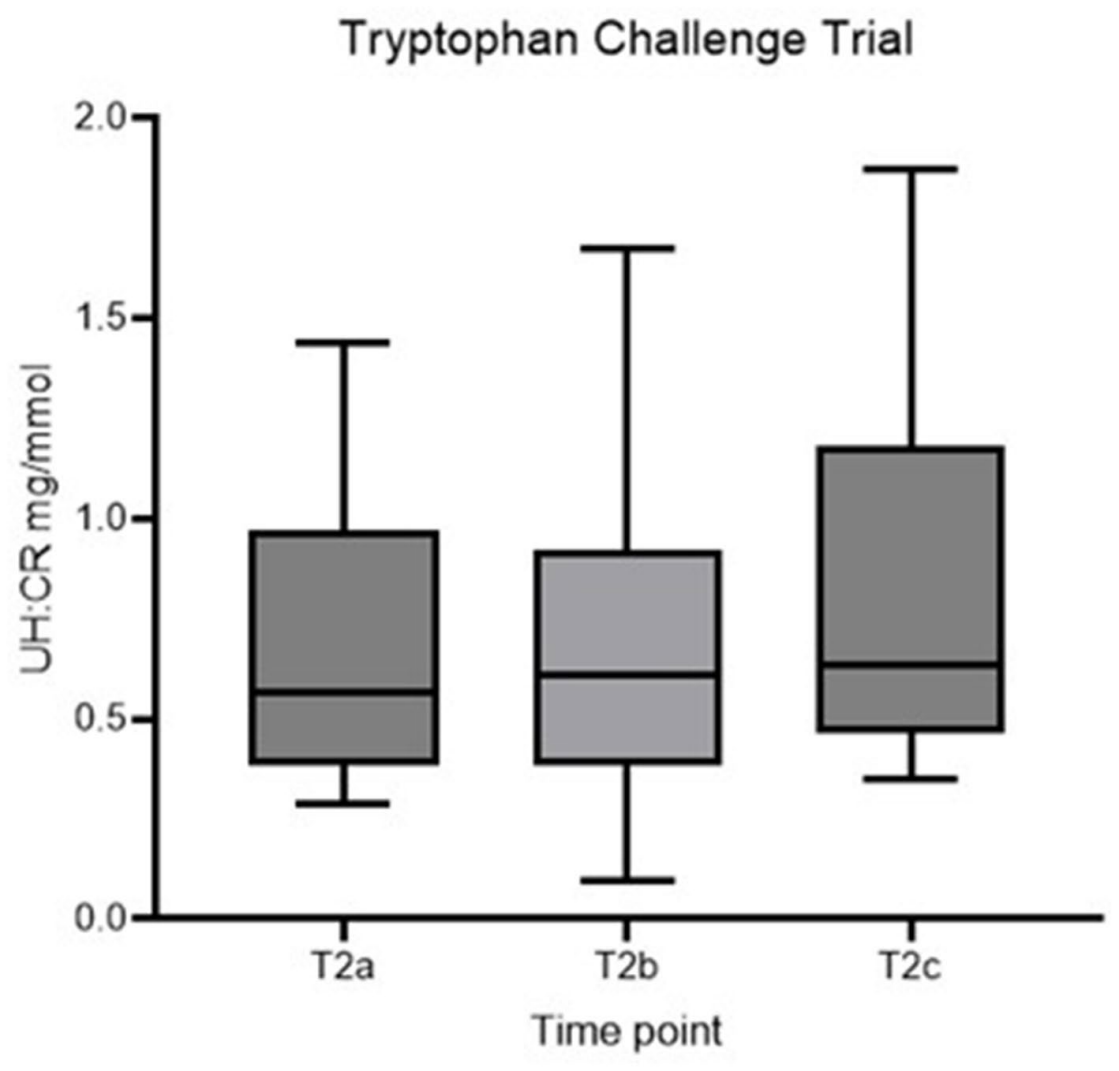
Box plot of UH: CR (mg/mmol) of healthy controls at T2a, T2b and T2c. UH: CR, urinary 5-HIAA-to-creatinine ratio.

**Figure 4:**
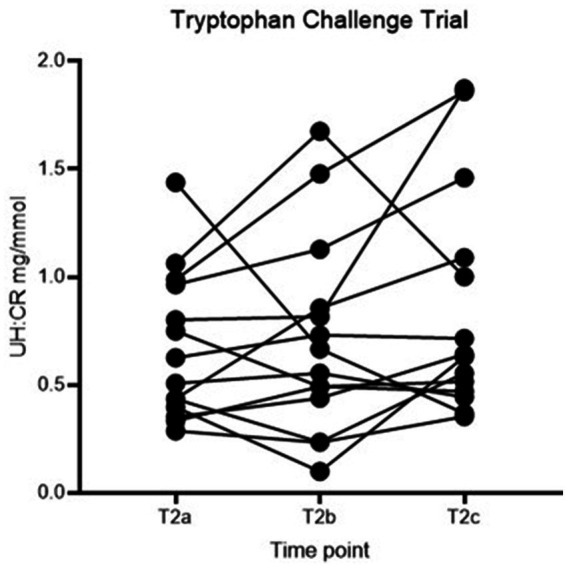
Before-after plot of UH: CR (mg/mmol) of healthy controls at T2a, T2b and T2c. UH: CR, urinary 5-HIAA-to-creatinine ratio.

No statistically significant difference in UH: CR was detected between the three time points (*p* > 0.05), and the second hypothesis is rejected (Figure 3; Table 3). However, individual dogs demonstrated variable response patterns, with some peaking at 4 h and others at 8 h. Figure 4 illustrates the before-after plot of UH: CR of all 14 dogs at their respective time points throughout the TRP challenge. 4/14 dogs peaked at T2_b_, the anticipated peak concentration on our hypothesis. Eight of 14 dogs peaked at T2_c_, 8 hafter the TRP challenge. Among the eight dogs, five showed a continuously increasing trend from T2_a_ through to T2_c_, while the remaining three dogs had a drop at T2_b_, 4 h after the TRP challenge from T2_a_, before increasing to the peak concentration at T2_c_.

**Table 3:** Friedman test result of tryptophan challenge between T2a, T2b and T2c.

Time points	n	*p-*value
T_2a_ VS T_2b_	14	>1
T_2a_ VS T_2c_	14	0.176
T_2b_ VS T_2c_	14	>1

n, number of dogs.

## Discussion

The main objective of this study was to validate a commercial ELISA assay for urinary 5-HIAA measurement in dogs and to investigate whether dietary TRP supplementation or a TRP challenge altered urinary 5-HIAA excretion. The assay was sufficiently linear and precise for use with canine urine when a dilution step was applied. Dietary TRP supplementation was associated with changes in urinary 5-HIAA-to-creatinine ratios and reduced variability between individuals, whereas an oral TRP challenge did not significantly alter urinary excretion of 5-HIAA within the study timeframe. The following sections discuss these findings in detail, including assay validation, the effects of TRP supplementation and TRP challenge, and the study limitations.

### Urine 5-HIAA assay validation

The IBL-America urinary 5-HIAA assay is sufficiently linear and precise for laboratory use with canine urine samples. However, accuracy (as reflected by quantitative spiking recovery) was marginal (overall average spiking recovery of 132.6%). Given the marked matrix effect observed, greater dilution of the canine urine samples may improve the assay’s accuracy. This strategy, however, may compromise assay sensitivity, particularly in healthy dog samples.

### The effect of dietary tryptophan supplementation on urinary 5-HIAA excretion

The second part of the study reported here aimed to evaluate the effect of TRP supplementation on serotonin concentration in healthy dogs. We compared the UH: CR at baseline, after dietary TRP supplementation, and after the washout period for the initial analysis. Interestingly, two statistically significant differences between timepoints were noted: between T_0_ and T_2a_, and between T_1_ and T_2a_. Between T_0_ and T_2a_, all 14 dogs received dietary tryptophan supplementation, dosed according to their respective weights. Throughout the trial, dogs were fed the same standardized diet. Dietary TRP appeared to be associated with a reduction in intra-group variability of UH: CR at T_2a_ compared to T_0_, as shown by the reduction in within-group CV (Table 2).

Tryptophan hydroxylase (TRH-1) is the rate-limiting enzyme for 5-HT/5-HIAA synthesis. We speculate that during T_1_, the daily increase in TRP exposure led to an increase in 5-HIAA synthesis in the 5-HT pathway. An increase in 5-HT/5-HIAA to more than the body’s requirement may lead to negative feedback on TRH-1, limiting further 5-HT synthesis. Joh (13) suggested that exposure to a prolonged high 5-HT concentration may reduce the gene expression of TRH-1—another mechanism for maintaining the 5-HT concentration at a homeostatic level. Plasma TRP concentration is tightly controlled under normal physiological conditions for protein and neurotransmitter synthesis to avoid the over-production of neurotransmitters (14). It is possible that the above-proposed mechanisms persisted for over 2 weeks, contributing to reduced 5-HIAA concentrations at the post-washout period and indirectly reducing the within-group variability at T_2a_ compared to T_0_.

The difference between T_1_ and T_2a_ was statistically significant, *p* = 0.042 (*p* < 0.05). UH: CR was considerably higher at T_1_ than at T_2a,_ likely due to the increased availability of TRP as the substrate for 5-HT/5-HIAA production. Dietary TRP supplementation may have exerted a sustained effect on TRP metabolism that persisted beyond the 2 weeks of the washout period. In one study assessing the long-term effect of methamphetamine on the metabolism of 5-HT in rat brains a marked reduction in 5-HT and 5-HIAA concentrations and TRH activity was reported, with these parameters slowly recovering over 10 days after cessation of the drug (15). Interestingly, TRH activity remained depressed in some specific regions in the brain for a prolonged period after the challenge. However, some conflicting evidence shows that chronic L-TRP supplementation at incrementing doses did not affect the urinary excretion of TRP metabolites, including 5-HIAA (16). To date, there is a lack of precise data to define the necessary timeframe for 5-HIAA excretion to return to normal after cessation of chronic TRP use.

Our first hypothesis was that UH: CR would be higher after TRP loading (T_1_) than at baseline (T_0_). Figure 2 shows increased intra-group variation at T_1_, with a higher mean UH: CR at T_1_ (0.970 ± 0.639 mg/mmol) than T_0_ (0.918 ± 0.483 mg/mmol). The difference between the two time points did not reach statistical significance and thus the hypothesis was rejected. One of the possible reasons for insignificant results was unstandardized environmental conditions for the 14 controls except for the diet. The controls were staff-owned dogs that live in various environmental conditions and have variable routines, e.g., mealtimes, exercise before/after mealtimes, exposure to environmental stressors, etc. The above may affect the level of stress experienced by the dogs and inevitably affect the 5-HT concentration as elevation in stress hormone cortisol increases 5-HT uptake, reducing the availability of circulating 5-HT (17). Stress-related variability may have influenced the measured urine 5-HIAA concentration. The study’s sample size was also small, with post-hoc statistical power calculations indicating that the study had only a 4.3% power for detecting a difference of this magnitude at an alpha probability of 5%. Using the data obtained to calculate group sizes necessary to achieve an 80% power yields a group size of 2,370/treatment arm. This is unlikely to be feasible in real-life situations. Other strategies to reduce variability, such as using HPLC–MS/MS or GC–MS/MS methodologies to decrease assay variability, may reduce these numbers. However, it is unlikely that this difference would be dramatic.

One of the major limitations of this study is inconsistent TRP dosing times at home. This might impact on the 5-HIAA concentration at collection. No standardized dosing guideline was provided regarding the TRP supplement being given before, with, or after meal dosing; hence, the presence/absence of food in the stomach may also affect the amount absorbed in the intestines. Carbohydrate-rich food increased the TRP to large neutral amino acids ratio of up to 14% above the baseline. In contrast, protein-rich food reduced the ratio by up to 35% below the baseline according to a human study investigating the effect of food composition on the TRP and tyrosine ratios (18). A study in pigs investigated the influence of different dietary proteins on plasma TRP concentrations. It showed that plasma TRP remained elevated for 4 h after ingesting an alpha-lactalbumin-containing meal (19). These strengthen the evidence that the presence of food can affect the TRP concentration at various time points. Future studies should incorporate a consistent feeding protocol to reduce this variability. Access to serotonin-rich food, certain fruits, e.g., banana, kiwifruit, pineapple, etc., can lead to significant elevation in serum and urine 5-HIAA with reported peak concentration at 2 h post-ingestion in serum (20, 21). Hence, a fasted sample is generally recommended prior to a 24-h urine 5-HIAA assay in humans, where the primary use of urine 5-HIAA is for detection and follow-up on cases of carcinoid tumors (21). To counteract this limitation, a serum 5-HIAA using liquid chromatography with tandem mass spectrometry was developed and has been supported as a more reliable and accurate means of analyzing 5-HIAA (22, 23).

### The effect of an oral tryptophan challenge on urinary 5-HIAA excretion

In the second part of the study reported here, we assessed urinary 5-HIAA excretion in healthy dogs in response to an oral tryptophan loading dose. In a human study assessing the acute effect of TRP challenge in healthy people and people with schizophrenia, plasma 5-HIAA showed a maximal increase at 240 min in both groups (24). The collection times were 30, 60, 90, and 240 min after the challenge; hence, the concentration might continue to rise post-study time, and it likely requires more extended time for urine concentration to rise. Another study showed peak serum 5-HIAA concentration at 120 min after ingesting 5-HT-rich food (21). Both doses of TRP used and time from administration play a role in the 5-HT/5-HIAA concentrations—5-HIAA peaked at 60 to 150 min when given at 50 mg/kg (25). With the above information, we proposed the second hypothesis that UH: CR would be higher than baseline following an oral TRP challenge and reach peak concentration 4 h post-challenge. No statistically significant differences were identified between the 3 time points in the TRP challenge (Table 3). The before-after plot in Figure 4 showed variable responses between individuals. 4/14 dogs peaked at T_2b_, the anticipated time to reach peak concentration, and 8/14 dogs reached their highest concentrations at T_2c_, over 50% of the number of healthy controls.

The other plausible explanation of the variable responses toward the TRP challenge were likely due to interindividual variations such as metabolic rate, anxiety levels, and genetic/phenotypic compositions that may impact the pharmacokinetics of the TRP supplement. A commercially available TRP supplement—Paw by Blackmores Complete Calm was selected for this phase of the study for translational relevance, as it reflects real-world owner supplementation practices; each chew contains 180 mg of TRP and a variety of micronutrients, e.g., vitamins A, B, and E and micro-minerals including zinc sulfate, selenium, manganese oxide, iron sulfate, etc. thus affecting the purity of the TRP supplementation and may indirectly affect the level of absorption due to the interactions between these substances and effect on the availability of TRP after metabolism which is another recognized limitation of the study. Future studies to more precisely define responses to oral tryptophan challenge, using pure L-tryptophan at a defined mg/kg dose, are warranted.

All dogs were dosed according to the recommended amount per the manufacturer’s guidelines. According to the National Research Council recommendations (2006), dogs require a minimum of 1.1 g of TRP per kg of dry matter, based on a dietary energy density of 4,000 kcal of ME per kg, to support their basic needs. The TRP requirements seemed to vary with different life stages and various breeds, with Beagles needing a higher TRP concentration than Labradors or Dachshunds (14, 26). The doses administered during the TRP challenge trial reported here may be insufficient to stimulate a consistent response in all dogs. We speculate that all the dogs would respond according to our second hypothesis if a higher dose of TRP were used. Notably, the dose administered to human subjects in the study reported by Sathyasaikumar et al. (24) was 6 grams/subject, assuming an average body weight of 75 kg, this represented approximately 80 mg/kg while the doses administered to the dogs in the challenge phase of the study reported here ranged from 25–70 mg/kg. Further studies of 5-HIAA response to TRP challenge in dogs at higher doses than used here are warranted, as a necessary step before assessing TRP challenge response in dogs with chronic enteropathies.

Another important limitation of this study is the absence of formal blinding and randomization. All dogs underwent supplementation and challenge phases sequentially, which may have introduced order effects, where residual influences of the first phase could carry over and affect the outcomes of the subsequent phase, despite the inclusion of a two-week washout. In addition, because investigators were not blinded to treatment phase, subtle biases in data collection and interpretation cannot be fully excluded. Randomizing treatment order and incorporating blinded assessment would strengthen the internal validity of future studies.

Furthermore, baseline hematology and serum biochemistry were not performed during recruitment. Although all dogs were judged to be clinically healthy on history and physical examination, subclinical conditions cannot be ruled out. Such conditions could alter TRP metabolism, 5-HT turnover, or creatinine excretion, introducing unrecognized variability into urinary 5-HIAA measurements. This reduces confidence that all enrolled dogs represented a uniformly healthy baseline cohort. Future studies should include standardized pre-screening to minimize this potential source of confounding.

## Conclusion

This study probed the relationship between oral TRP supplementation and urinary 5-HIAA excretion as an indicator of 5-HT metabolism in healthy dogs. The data obtained suggest that TRP supplementation reduces the intra-individual variability between healthy dogs, possibly due to downregulation of TRH-1 activity or the surge of TRP concentration promoting negative feedback onto TRH-1. The lack of statistically significant differences between UH: CR at the 3-time points at the TRP challenge may reflect inadequate dosing in this pilot study, and possibly the short sampling period. Further work in this area is necessary.

A proposed future project is to compare the 5-HT concentrations between healthy dogs and dogs with CE undergoing tryptophan loading/challenge trials, as there is building evidence that 5-HT dysfunction may play a role in the pathogenesis of CE in humans. The inclusion of clinical scores, e.g., CIBDAI or CCECAI, to monitor the clinical severity of dogs with CE on TRP loading/challenge trials would add validity to the study results in addition to the serotonin concentrations. Future studies should also integrate measurement of serum 5-HIAA alongside urinary assays, as serum-based methods are less influenced by dietary 5-HT intake and there may provide a more precise reflection of systemic 5-HT metabolism. TRP supplementation or SSRIs may be considered therapeutic means for dogs with CE upon further investigation.

## Data Availability

The original contributions presented in the study are included in the article/supplementary material, further inquiries can be directed to the corresponding author.
